# Supplementary material for: Heterogeneous changes of soil microclimate in high mountains and glacier forelands
Source: Nat Commun. 2023 Aug 31;14:5306. doi: 10.1038/s41467-023-41063-6 (PMC10471727; doi:10.1038/s41467-023-41063-6)
Supplement: Supplementary file 1 — Supplementary information [file 41467_2023_41063_MOESM1_ESM.pdf]

Supplementary Information for

Heterogeneous changes of soil microclimate in high mountains and glacier forelands

Silvio Marta, Anaïs Zimmer, Marco Caccianiga, Mauro Gobbi, Roberto Ambrosini, Roberto Sergio Azzoni, Fabrizio Gili, Francesca Pittino, Wilfried Thuiller, Antonello Provenzale & Gentile Francesco Ficetola

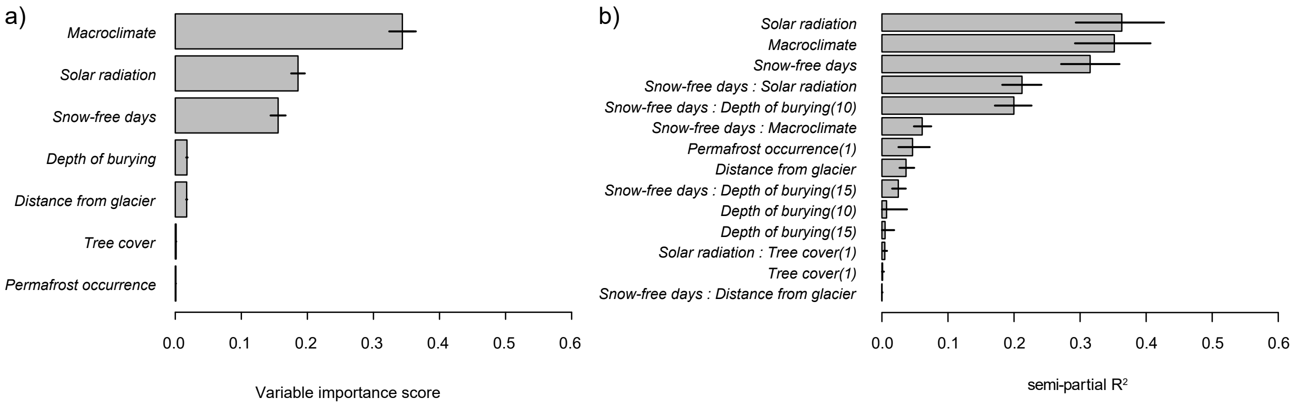

Supplementary Figure 1: Variable contribution to the full model in terms of **a)** variable importance score (single predictors, measuring the joint contribution to both additive and interactive terms) and **b)** semi-partial R^2 (single terms). Error bars represent the 95% confidence intervals for the average estimate, obtained with 1,000 randomizations for each predictor (a) or 1,000 bootstrap replicates (b). Source data are provided as a Source Data file.

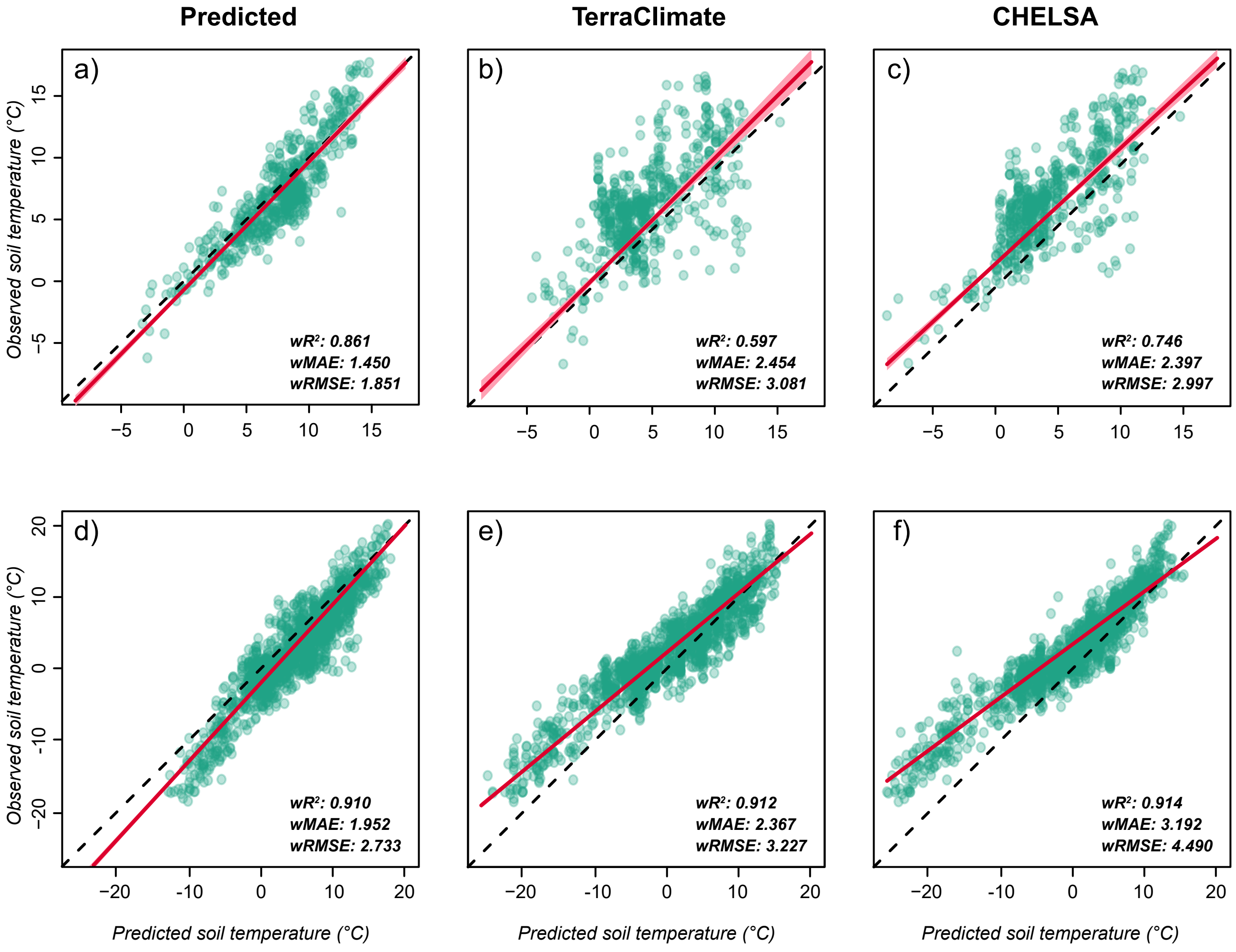

Supplementary Figure 2: Comparison between the performance of our model and alternative approaches to the estimation of local temperature. Local temperatures are estimated for both the training set (**a-c**) and the independent validation set (**d-f**). For each dataset, the recorded soil temperature was regressed against the predictions of our model, obtained using the coefficients from the leave-one-out analysis (**a, d**); the estimates obtained with the widely used climate products TerraClimate (**b, e**) and CHELSA (**c, f**). Due to reduced temporal extent of the CHELSA dataset, observations from 2020 and 2021 were excluded from all comparisons. The dashed black lines mark the perfect fit (1:1 line), while the red lines represent the fits from the weighted linear regression; shaded red areas represent the 95% confidence interval of the average estimates. To evaluate performances, the weighted coefficient of determination (wR^2), mean absolute error (*wMAE* in °C) and root mean square error (*wRMSE* in °C) are provided. Source data are provided as a Source Data file.

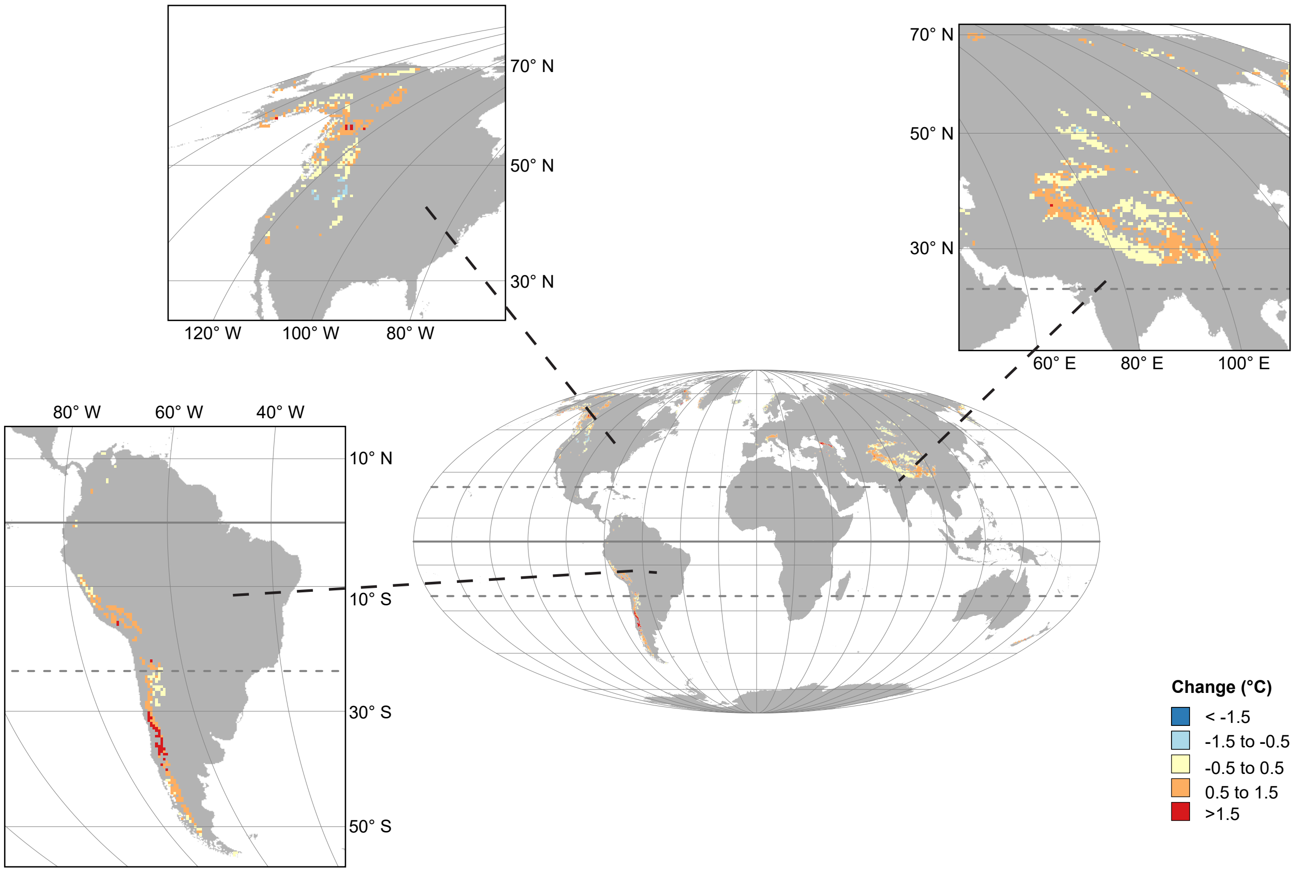

Supplementary Figure 3: Microclimate changes between 2016-2020 and 2001-2005 (cf. Figure 3a), highlighting changes in key mountain regions. The map reports per-cell average changes in soil temperature during the snow-free season; the dashed horizontal lines identify the Tropics, while the continuous tick one the Equator. Map projection: Mollweide (ESRI:54009); grid: 20*20 degrees; resolution: 50 km. Source data are provided as a Source Data file.

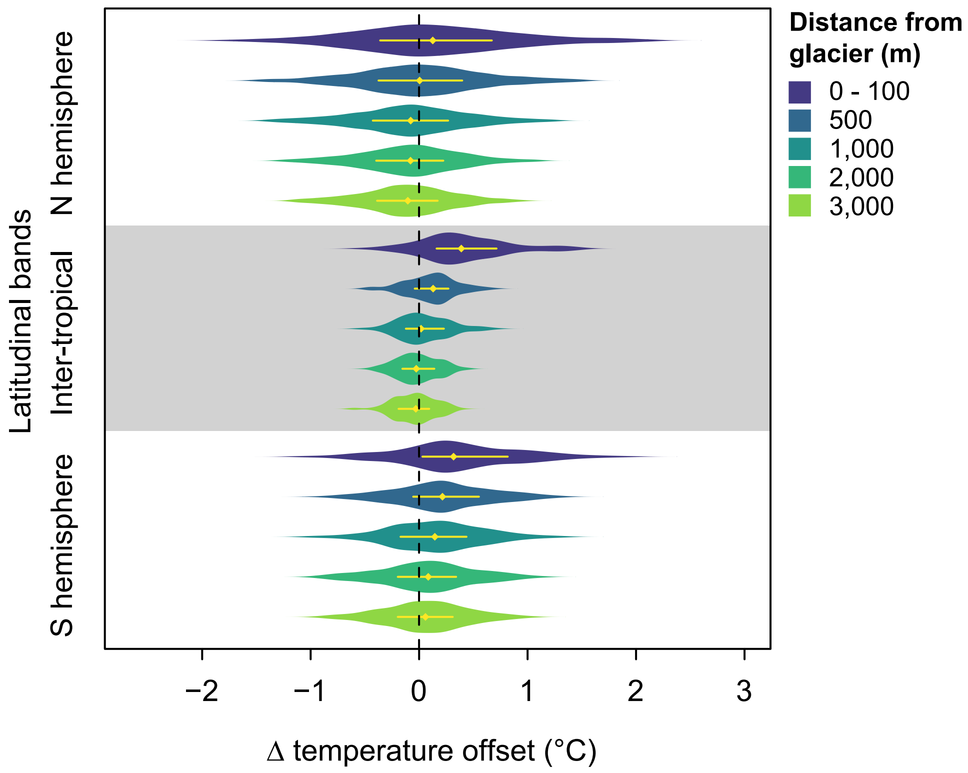

Supplementary Figure 4: Microclimate changes between 2016-2020 and 2001-2005 (cf. Figure 3b). Violin plots summarizing the trends of temperature offsets for each distance class and latitudinal band during the snow-free season. Offsets were calculated as the difference between the modelled soil temperature and macroclimate (monthly air temperature from TerraClimate) during the months with percentage of snow-free days > 20%, and averaging monthly estimates over the entire snow-free season. temperature offsets were obtained by subtracting the 2001-2005 offset from the 2016-2020 offset. Positive values thus indicate that, during 2001-2020, microclimate change is larger than the macroclimate change. Yellow dots mark the median value for each series, while yellow lines the first and third quartiles. Source data are provided as a Source Data file.

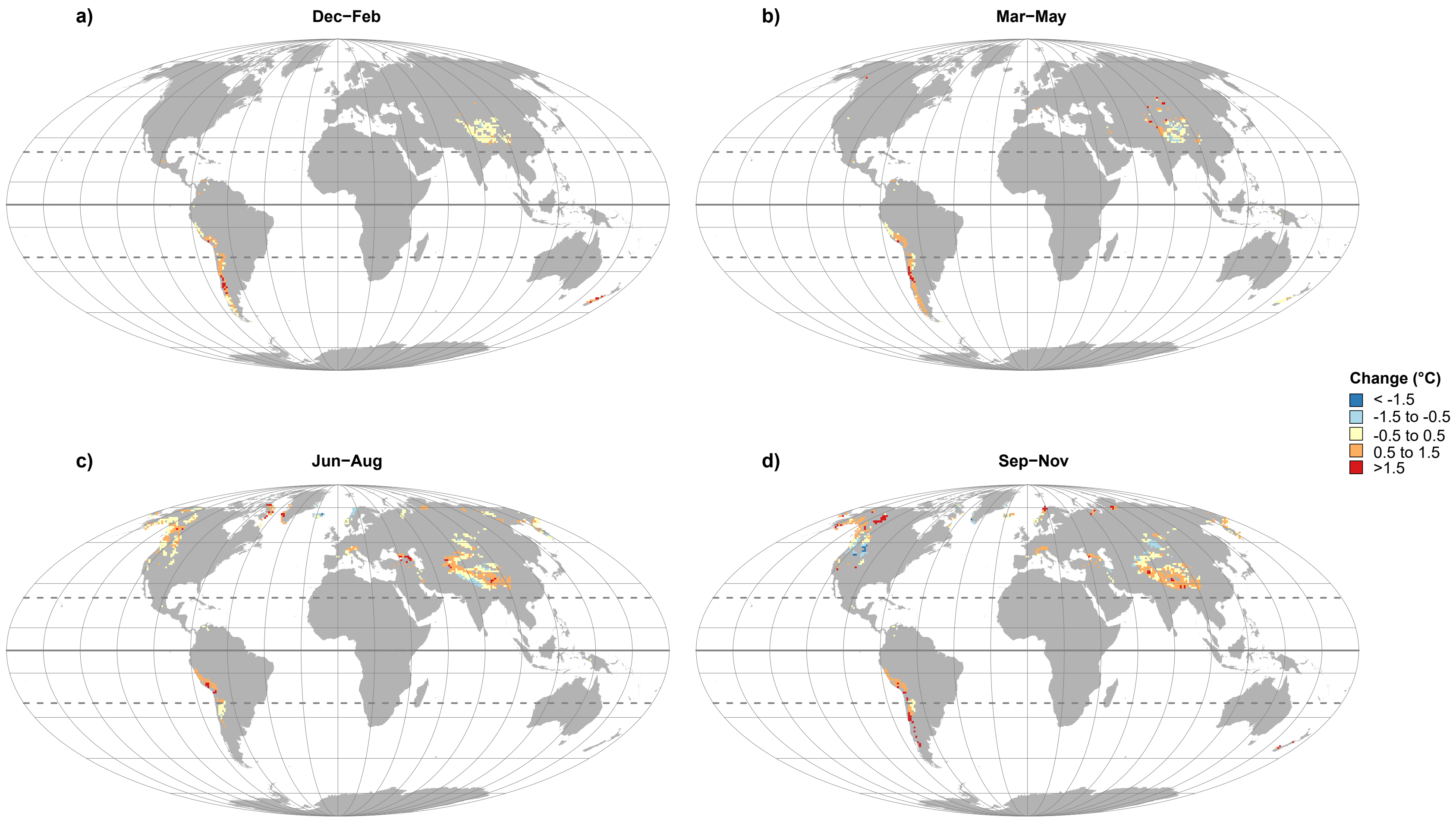

Supplementary Figure 5: Seasonal trends of temperature change between 2016-2020 and 2001-2005. Per-cell average changes in soil temperature during **a)** December-February, **b)** March-May, **c)** June-August and **d)** September-November. In each map, only cells with percentage of snow-free days > 20% are considered; the dashed horizontal lines identify the Tropics, while the continuous tick line indicates the Equator. Map projection: Mollweide (ESRI:54009); grid: 20*20 degrees; resolution: 100 km. Source data are provided as a Source Data file.

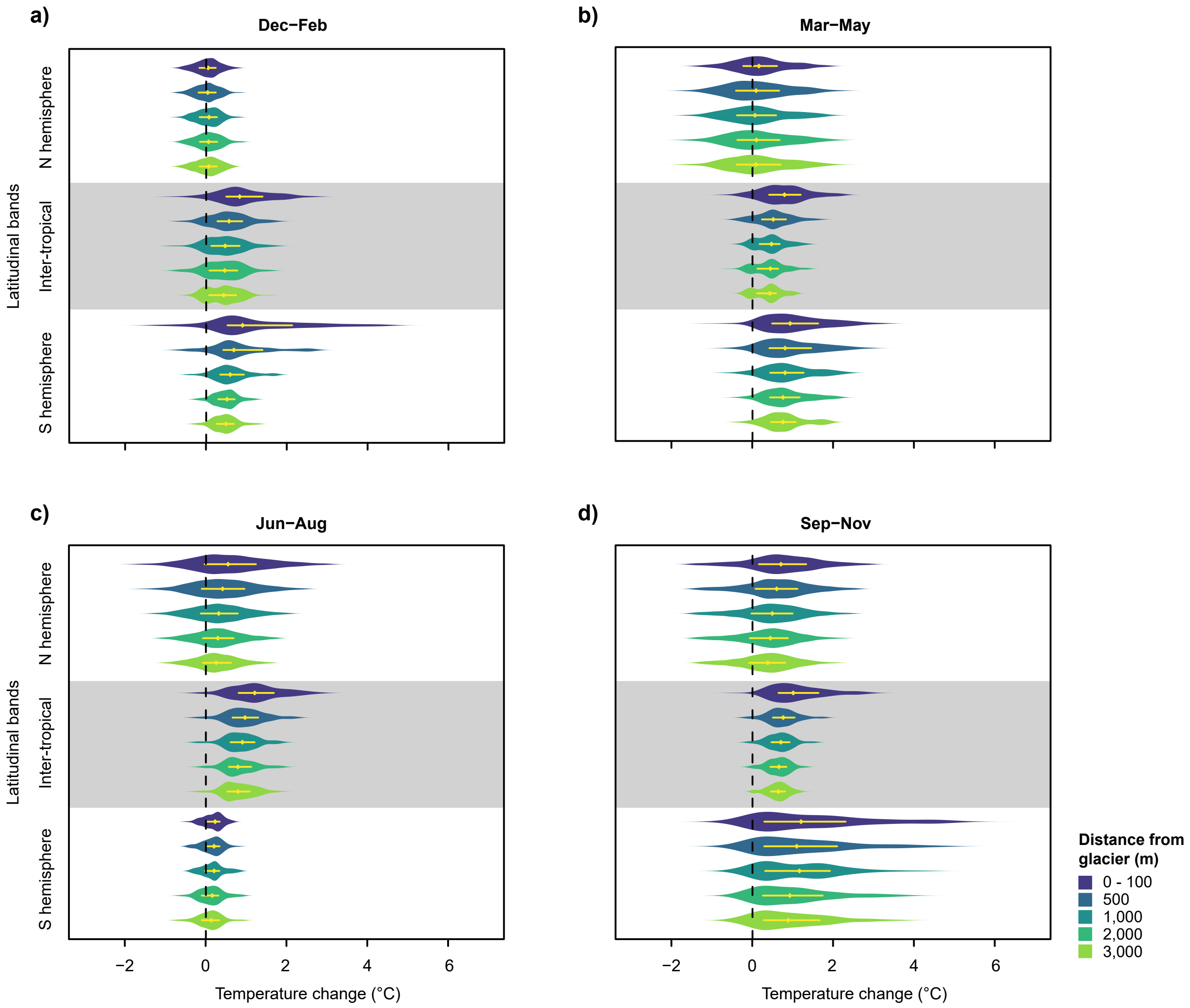

Supplementary Figure 6: Seasonal trends of temperature change between the periods 2016-2020 and 2001-2005. For each latitudinal band and distance class, violin plots summarize temperature changes in soil temperature during **a)** December-February, **b)** March-May, **c)** June-August and **d)** September-November. Yellow dots mark the median value for each series, while yellow lines the first and third quartiles. Source data are provided as a Source Data file.

Supplementary Table 1: Summary table for fixed effects of the linear mixed model used to reconstruct soil temperature. Estimated coefficients (β estimated) and standard errors (β SE) are reported, together with a type III analysis of variance table for single terms, with degrees of freedom calculated with the Satterthwaite's method. Source data are provided as a Source Data file.

	β estimated	β SE	df (num, den)	F	p-value
Intercept	8.633	0.382			
mT	1.677	0.068	1, 1478.47	605.82	< 0.0001
sfd	1.590	0.107	1, 1499.47	289.37	< 0.0001
rad	1.364	0.066	1, 1490.47	63.69	< 0.0001
dg	0.394	0.042	1, 1500.93	86.27	< 0.0001
pf	-0.923	1.052	1, 24.17	0.77	0.389
tc	-0.973	0.401	1, 1488.23	5.89	0.015
d			2, 44.41	14.44	< 0.0001
d(10)	-1.064	0.578			
d(15)	-1.961	0.369			
sfd:mT	0.156	0.042	1, 1489.82	13.93	0.0002
sfd:rad	0.180	0.044	1, 1483.71	16.76	< 0.0001
sfd:dg	-0.020	0.039	1, 1496.57	0.25	0.614
tc:rad	-0.379	0.271	1, 1483.33	1.97	0.161
d:sfd			2, 1499.40	12.74	< 0.0001
d(10):sfd	-0.366	0.111			
d(15):sfd	-0.798	0.159			

mT: macroclimate (downscaled monthly average temperature), rad: daily cumulative shortwave solar radiation, sfd: monthly frequency of snow-free days, dg: distance from glacier forefront, tc: tree cover, pf: permafrost occurrence, and d: depth of burying (10 or 15 cm below soil surface).

Supplementary Table 2: Summary statistics for the coefficients of the linear mixed model used to reconstruct soil temperature, estimated using the leave-one-out analysis. Mean, standard deviation (σ), minimum, maximum and interquartile range (IQR; i.e. Q3-Q1). Source data are provided as a Source Data file.

	mean	σ	minimum	maximum	IQR
Intercept	8.634	0.091	8.449	8.807	0.129
mT	1.677	0.044	1.612	1.807	0.029
sfd	1.587	0.040	1.483	1.675	0.032
rad	1.365	0.020	1.320	1.405	0.018
dg	0.395	0.024	0.347	0.460	0.010
pf	-0.926	0.242	-1.805	-0.212	0.123
tc	-0.991	0.254	-1.784	-0.130	0.057
d(10)	-1.065	0.123	-1.258	-0.753	0.141
d(15)	-1.962	0.155	-2.359	-1.381	0.039
sfd:mT	0.157	0.012	0.124	0.183	0.008
sfd:rad	0.179	0.013	0.147	0.208	0.011
sfd:dg	-0.018	0.016	-0.046	0.015	0.010
tc:rad	-0.352	0.182	-0.558	0.494	0.051
d(10):sfd	-0.363	0.046	-0.461	-0.237	0.028
d(15):sfd	-0.794	0.068	-0.912	-0.573	0.072

mT: macroclimate (downscaled monthly average temperature), rad: daily cumulative shortwave solar radiation, sfd: monthly frequency of snow-free days, dg: distance from glacier forefront, tc: tree cover, pf: permafrost occurrence, and d: depth of burying (10 or 15 cm below soil surface).

Supplementary Table 3: Geographic attributes, device model, recording parameters and burying depth for the 175 sampling sites analysed.

Plotcode	Region	Glacier	Longitude (wgs84)	Latitude (wgs84)	Elevation (m)	Device	Model	Start time	Stop time	Frequency (hh)	Records (n)	Depth (cm)
FERDI1960C	Polar	Ferdinandbreen	16.3756	78.71324	219	iButton	DS1923	20/07/2018 04:00	26/06/2019 08:00	4	2048	5
FERDI1990B	Polar	Ferdinandbreen	16.35597	78.71059	229	iButton	DS1923	20/07/2018 04:00	26/06/2019 08:00	4	2048	5
FERDI2009B	Polar	Ferdinandbreen	16.34191	78.71156	257	iButton	DS1923	20/07/2018 04:00	26/06/2019 08:00	4	2048	5
PLATA1936B	Polar	Platabreen	15.48802	78.20024	532	iButton	DS1923	20/07/2018 04:00	26/06/2019 08:00	4	2048	5
PLATA1948B	Polar	Platabreen	15.47541	78.20056	541	iButton	DS1923	20/07/2018 04:00	26/06/2019 08:00	4	2048	5
PLATA1990A	Polar	Platabreen	15.47353	78.19811	545	iButton	DS1923	20/07/2018 04:01	26/06/2019 08:01	4	2048	5
AGO_M1	Mid-latitudes	Agola	10.8583	46.15178	2613	Tinytag Plus 2	TGP-4500	18/07/2018 00:00	29/08/2019 00:00	1	9769	15
AGO_M2	Mid-latitudes	Agola	10.85397	46.15273	2551	Tinytag Plus 2	TGP-4500	18/07/2018 00:00	29/08/2019 00:00	1	9769	15
AGO_M3	Mid-latitudes	Agola	10.85007	46.15405	2483	Tinytag Plus 2	TGP-4500	18/07/2018 00:00	29/08/2019 00:00	1	9769	15
AGO_M4	Mid-latitudes	Agola	10.85747	46.152	2601	Tinytag Plus 2	TGP-4500	18/07/2018 00:01	29/08/2019 00:01	1	9769	15
AGO_M5	Mid-latitudes	Agola	10.85178	46.15305	2524	Tinytag Plus 2	TGP-4500	18/07/2018 00:00	29/08/2019 00:00	1	9769	15
AGO_M6	Mid-latitudes	Agola	10.85712	46.15268	2610	Tinytag Plus 2	TGP-4500	18/07/2018 00:01	29/08/2019 00:01	1	9769	15
AGOLA1973A	Mid-latitudes	Agola	10.85676	46.1524	2585	iButton	DS1923	29/08/2018 04:00	15/07/2019 08:00	4	1922	5
AGOLA1994B	Mid-latitudes	Agola	10.85708	46.15178	2600	iButton	DS1923	29/08/2018 04:00	15/07/2019 08:00	4	1922	5
AMO_DC	Mid-latitudes	Amola	10.68872	46.21803	2637	Tinytag Plus 2	TGP-4500	15/07/2011 00:00	15/07/2012 22:00	2	4404	15
AMO_PEG	Mid-latitudes	Amola	10.70553	46.21403	2459	Tinytag Plus 2	TGP-4500	05/08/2011 00:28	05/08/2012 23:08	1	8808	15
AMOLA2003C	Mid-latitudes	Amola	10.69425	46.21672	2569	iButton	DS1923	25/08/2018 04:00	01/08/2019 08:00	4	2048	5
AMOLAPEGB	Mid-latitudes	Amola	10.70384	46.21328	2464	iButton	DS1923	25/08/2018 04:00	01/08/2019 08:00	4	2048	5
CLA_RG3	Mid-latitudes	Clapier	7.41897	44.12234	2610	TinyTag Plus	TGP-4500	31/07/2019 23:00	15/09/2020 23:00	0.5	18347	15
CLA_RG5	Mid-latitudes	Clapier	7.42306	44.11403	2833	TinyTag Plus	TGP-4500	01/08/2019 00:00	15/09/2020 23:00	0.5	18351	15
DAMMA1946A	Mid-latitudes	Dammagletscher	8.46104	46.63856	2028	iButton	DS1923	19/07/2018 04:00	25/06/2019 08:00	4	2048	5
DAMMA1992A	Mid-latitudes	Dammagletscher	8.45995	46.63666	2052	iButton	DS1923	19/07/2018 04:01	25/06/2019 08:01	4	2048	5
DAMMA2003C	Mid-latitudes	Dammagletscher	8.45873	46.63577	2075	iButton	DS1923	19/07/2018 04:00	25/06/2019 08:00	4	2048	5
FORNI1850	Mid-latitudes	Forni	10.56628	46.41984	2184	iButton	DS1923	26/07/2018 04:01	02/07/2019 08:01	4	2048	5
FORNI1850b	Mid-latitudes	Forni	10.55756	46.41842	2162	iButton	DS1923	26/07/2018 04:00	02/07/2019 08:00	4	2048	5
FORNI1926	Mid-latitudes	Forni	10.5684	46.41863	2188	iButton	DS1923	26/07/2018 04:00	02/07/2019 08:00	4	2048	5
FORNI1945	Mid-latitudes	Forni	10.57401	46.41648	2220	iButton	DS1923	26/07/2018 04:00	02/07/2019 08:00	4	2048	5
FORNI1955	Mid-latitudes	Forni	10.57964	46.41221	2312	iButton	DS1923	26/07/2018 04:00	02/07/2019 08:00	4	2048	5

FORNI2000	Mid-latitudes	Forni	10.5851	46.40689	2503	iButton	DS1923	26/07/2018 04:00	02/07/2019 08:00		4	2048	5
B1_01	Mid-latitudes	Glacier Blanc-Noir	6.40521	44.93746	2485	HOBO	8K Pendant	24/07/2019 05:00	26/07/2020 01:00		4	2208	10
B1_02	Mid-latitudes	Glacier Blanc-Noir	6.40513	44.93811	2501	HOBO	8K Pendant	24/07/2019 05:00	26/07/2020 01:00		4	2208	10
B1_03	Mid-latitudes	Glacier Blanc-Noir	6.40537	44.93715	2473	HOBO	8K Pendant	31/07/2019 17:00	26/07/2020 10:00		1	8658	10
B2_01	Mid-latitudes	Glacier Blanc-Noir	6.40735	44.93445	2350	HOBO	8K Pendant	24/07/2019 05:00	26/07/2020 05:00		4	2209	10
B2_02	Mid-latitudes	Glacier Blanc-Noir	6.40735	44.93445	2350	HOBO	8K Pendant	24/07/2019 05:00	26/07/2020 05:00		4	2209	10
B3_01	Mid-latitudes	Glacier Blanc-Noir	6.4097	44.93359	2318	HOBO	8K Pendant	24/07/2019 05:00	26/07/2020 05:00		4	2209	10
B3_02	Mid-latitudes	Glacier Blanc-Noir	6.4097	44.93359	2318	HOBO	8K Pendant	24/07/2019 05:00	26/07/2020 05:00		4	2209	10
B4_01	Mid-latitudes	Glacier Blanc-Noir	6.40879	44.93075	2311	HOBO	8K Pendant	24/07/2019 05:00	26/07/2020 09:00		4	2210	10
B4_02	Mid-latitudes	Glacier Blanc-Noir	6.40879	44.93075	2311	HOBO	8K Pendant	24/07/2019 05:00	26/07/2020 09:00		4	2210	10
BLIA_X	Mid-latitudes	Glacier Blanc-Noir	6.41403	44.93242	2457	HOBO	8K Pendant	24/07/2019 05:00	26/07/2020 05:00		4	2209	10
BLIA_Y	Mid-latitudes	Glacier Blanc-Noir	6.41336	44.93381	2447	HOBO	8K Pendant	24/07/2019 05:00	26/07/2020 05:00		4	2209	10
G1_01	Mid-latitudes	Glacier de Gebroulaz	6.62527	45.30892	2645	HOBO	8K Pendant	01/08/2019 13:00	18/07/2021 21:00		4	4305	10
G1_02	Mid-latitudes	Glacier de Gebroulaz	6.62406	45.30959	2630	HOBO	8K Pendant	01/08/2019 13:00	18/07/2021 21:00		4	4305	10
G1_03	Mid-latitudes	Glacier de Gebroulaz	6.622	45.31143	2593	HOBO	8K Pendant	01/08/2019 13:00	18/07/2021 21:00		4	4305	10
G2_01	Mid-latitudes	Glacier de Gebroulaz	6.6266	45.30866	2659	HOBO	8K Pendant	01/08/2019 13:00	18/07/2021 21:00		4	4305	10
G2_02	Mid-latitudes	Glacier de Gebroulaz	6.62749	45.30809	2664	HOBO	8K Pendant	01/08/2019 13:00	18/07/2021 21:00		4	4305	10
G2_03	Mid-latitudes	Glacier de Gebroulaz	6.62867	45.30645	2677	HOBO	8K Pendant	01/08/2019 13:00	18/07/2021 21:00		4	4305	10
G3_01	Mid-latitudes	Glacier de Gebroulaz	6.62424	45.31068	2617	HOBO	8K Pendant	01/08/2019 13:00	18/07/2021 21:00		4	4305	10
G3_02	Mid-latitudes	Glacier de Gebroulaz	6.62807	45.30782	2671	HOBO	8K Pendant	01/08/2019 13:00	18/07/2021 21:00		4	4305	10
G3_03	Mid-latitudes	Glacier de Gebroulaz	6.6308	45.30311	2737	HOBO	8K Pendant	01/08/2019 13:00	18/07/2021 21:00		4	4305	10
GLIA_01	Mid-latitudes	Glacier de Gebroulaz	6.62368	45.31372	2584	HOBO	8K Pendant	01/08/2019 13:00	18/07/2021 21:00		4	4305	10
GLIA_02	Mid-latitudes	Glacier de Gebroulaz	6.62431	45.31657	2507	HOBO	8K Pendant	01/08/2019 13:00	18/07/2021 21:00		4	4305	10
GLIA_03	Mid-latitudes	Glacier de Gebroulaz	6.62473	45.3203	2417	HOBO	8K Pendant	01/08/2019 13:00	18/07/2021 21:00		4	4305	10
S1_01	Mid-latitudes	Glacier de Saint Sorlin	6.16762	45.16724	2722	HOBO	8K Pendant	23/07/2020 13:00	25/07/2021 21:00		4	2205	10
S1_02	Mid-latitudes	Glacier de Saint Sorlin	6.16763	45.16902	2699	HOBO	8K Pendant	23/07/2020 13:00	25/07/2021 21:00		4	2205	10
S1_03	Mid-latitudes	Glacier de Saint Sorlin	6.16601	45.1705	2702	HOBO	8K Pendant	23/07/2020 13:00	25/07/2021 21:00		4	2205	10
S2_01	Mid-latitudes	Glacier de Saint Sorlin	6.16638	45.17214	2669	HOBO	8K Pendant	23/07/2020 13:00	25/07/2021 21:00		4	2205	10
S2_02	Mid-latitudes	Glacier de Saint Sorlin	6.17223	45.16734	2689	HOBO	8K Pendant	23/07/2020 13:00	25/07/2021 21:00		4	2205	10
S3_01	Mid-latitudes	Glacier de Saint Sorlin	6.16623	45.17341	2698	HOBO	8K Pendant	23/07/2020 13:00	25/07/2021 21:00		4	2205	10
S3_02	Mid-latitudes	Glacier de Saint Sorlin	6.16624	45.17341	2698	HOBO	8K Pendant	23/07/2020 13:00	25/07/2021 21:00		4	2205	10
S3_03	Mid-latitudes	Glacier de Saint Sorlin	6.17228	45.16802	2687	HOBO	8K Pendant	23/07/2020 13:00	25/07/2021 21:00		4	2205	10
SLIA_01	Mid-latitudes	Glacier de Saint Sorlin	6.16957	45.17849	2611	HOBO	8K Pendant	23/07/2020 13:00	25/07/2021 21:00		4	2205	10

SLIA_02	Mid-latitudes	Glacier de Saint Sorlin	6.18201	45.17638	2513	HOBO	8K Pendant	23/07/2020 13:00	25/07/2021 21:00	4	2205	10
P1_01	Mid-latitudes	Glacier des Pelerins	6.8831	45.89477	2272	HOBO	8K Pendant	14/07/2019 17:00	02/08/2021 21:00	4	4502	10
P1_02	Mid-latitudes	Glacier des Pelerins	6.88393	45.8949	2285	HOBO	8K Pendant	14/07/2019 17:00	02/08/2021 21:00	4	4502	10
P2_01	Mid-latitudes	Glacier des Pelerins	6.88356	45.89672	2263	HOBO	8K Pendant	14/07/2019 17:00	02/08/2021 21:00	4	4502	10
P2_02	Mid-latitudes	Glacier des Pelerins	6.88358	45.89672	2263	HOBO	8K Pendant	14/07/2019 17:00	02/08/2021 21:00	4	4502	10
P2_03	Mid-latitudes	Glacier des Pelerins	6.88265	45.89589	2255	HOBO	8K Pendant	14/07/2019 17:00	02/08/2021 21:00	4	4502	10
P4_01	Mid-latitudes	Glacier des Pelerins	6.88244	45.89787	2204	HOBO	8K Pendant	14/07/2019 17:00	02/08/2021 21:00	4	4502	10
P4_02	Mid-latitudes	Glacier des Pelerins	6.88194	45.89812	2192	HOBO	8K Pendant	14/07/2019 17:00	02/08/2021 21:00	4	4502	10
P4_03	Mid-latitudes	Glacier des Pelerins	6.88194	45.89811	2192	HOBO	8K Pendant	14/07/2019 17:00	02/08/2021 21:00	4	4502	10
PLIA_01	Mid-latitudes	Glacier des Pelerins	6.87936	45.89827	2105	HOBO	8K Pendant	14/07/2019 17:00	02/08/2021 21:00	4	4502	10
PLIA_02	Mid-latitudes	Glacier des Pelerins	6.87934	45.89827	2105	HOBO	8K Pendant	14/07/2019 17:00	02/08/2021 21:00	4	4502	10
O1_01	Mid-latitudes	Glacier d'Orny	7.0683	46.00051	2671	HOBO	8K Pendant	01/09/2020 00:00	24/08/2021 20:00	4	2148	10
O1_02	Mid-latitudes	Glacier d'Orny	7.06828	46.00029	2678	HOBO	8K Pendant	01/09/2020 00:00	24/08/2021 20:00	4	2148	10
O2_01	Mid-latitudes	Glacier d'Orny	7.07	46.00009	2664	HOBO	8K Pendant	01/09/2020 00:00	24/08/2021 20:00	4	2148	10
O3_01	Mid-latitudes	Glacier d'Orny	7.07045	46.00048	2653	HOBO	8K Pendant	01/09/2020 00:00	24/08/2021 20:00	4	2148	10
O3_02	Mid-latitudes	Glacier d'Orny	7.07045	46.00048	2653	HOBO	8K Pendant	01/09/2020 00:00	24/08/2021 20:00	4	2148	10
O3_03	Mid-latitudes	Glacier d'Orny	7.07066	46.00006	2669	HOBO	8K Pendant	01/09/2020 00:00	24/08/2021 20:00	4	2148	10
O4_01	Mid-latitudes	Glacier d'Orny	7.07129	46.00022	2655	HOBO	8K Pendant	01/09/2020 00:00	24/08/2021 20:00	4	2148	10
O4_02	Mid-latitudes	Glacier d'Orny	7.07129	46.00022	2655	HOBO	8K Pendant	01/09/2020 00:00	24/08/2021 20:00	4	2148	10
O4_03	Mid-latitudes	Glacier d'Orny	7.07123	46.00089	2648	HOBO	8K Pendant	01/09/2020 00:00	24/08/2021 20:00	4	2148	10
O5_01	Mid-latitudes	Glacier d'Orny	7.07214	46.00075	2630	HOBO	8K Pendant	01/09/2020 00:00	24/08/2021 20:00	4	2148	10
O5_02	Mid-latitudes	Glacier d'Orny	7.07214	46.00075	2630	HOBO	8K Pendant	01/09/2020 00:00	24/08/2021 20:00	4	2148	10
O5_03	Mid-latitudes	Glacier d'Orny	7.07252	46.00059	2622	HOBO	8K Pendant	01/09/2020 00:00	24/08/2021 20:00	4	2148	10
T1_01	Mid-latitudes	Glacier du Tour	6.98341	45.9974	2583	HOBO	8K Pendant	14/07/2019 17:00	06/08/2021 23:00	4	4525	10
T1_02	Mid-latitudes	Glacier du Tour	6.98025	45.99847	2505	HOBO	8K Pendant	14/07/2019 17:00	06/08/2021 21:00	4	4526	10
T1_03	Mid-latitudes	Glacier du Tour	6.98064	45.99878	2487	HOBO	8K Pendant	14/07/2019 17:00	06/08/2021 23:00	4	4524	10
T2_01	Mid-latitudes	Glacier du Tour	6.9798	45.99921	2465	HOBO	8K Pendant	14/07/2019 17:00	06/08/2021 21:00	4	4526	10
T2_02	Mid-latitudes	Glacier du Tour	6.97977	45.99922	2465	HOBO	8K Pendant	06/07/2019 17:00	06/08/2021 21:00	4	4574	10
T2_03	Mid-latitudes	Glacier du Tour	6.97878	45.9994	2437	HOBO	8K Pendant	14/07/2019 17:00	06/08/2021 21:00	4	4526	10
T3_01	Mid-latitudes	Glacier du Tour	6.9769	46.00015	2370	HOBO	8K Pendant	14/07/2019 17:00	06/08/2021 21:00	4	4526	10
T3_02	Mid-latitudes	Glacier du Tour	6.97693	46.00016	2370	HOBO	8K Pendant	14/07/2019 17:00	06/08/2021 21:00	4	4526	10
T3_03	Mid-latitudes	Glacier du Tour	6.97955	45.99932	2452	HOBO	8K Pendant	14/07/2019 17:00	06/08/2021 21:00	4	4526	10
TLIA_01	Mid-latitudes	Glacier du Tour	6.95372	46.00094	1532	HOBO	8K Pendant	14/07/2019 17:00	06/08/2021 21:00	4	4526	10

MORTE1900C	Mid-latitudes	Morteratsch	9.94009	46.44563	1932	iButton	DS1923	19/07/2018 04:00	25/06/2019 08:00	4	2048	5
ODENW1850A	Mid-latitudes	Odenwinkelkees	12.63534	47.13102	2075	iButton	DS1923	28/07/2018 04:00	04/07/2019 08:00	4	2048	5
ODENW1890E	Mid-latitudes	Odenwinkelkees	12.63446	47.12916	2081	iButton	DS1923	28/07/2018 04:01	04/07/2019 08:01	4	2048	5
ODENW1931D	Mid-latitudes	Odenwinkelkees	12.63486	47.1278	2096	iButton	DS1923	28/07/2018 04:00	04/07/2019 08:00	4	2048	5
ODENW1969B	Mid-latitudes	Odenwinkelkees	12.6381	47.12429	2123	iButton	DS1923	28/07/2018 04:00	04/07/2019 08:00	4	2048	5
ODENW1985A	Mid-latitudes	Odenwinkelkees	12.63973	47.12209	2162	iButton	DS1923	28/07/2018 04:01	04/07/2019 08:01	4	2048	5
ODENW2008A	Mid-latitudes	Odenwinkelkees	12.64013	47.12084	2175	iButton	DS1923	28/07/2018 04:00	04/07/2019 08:00	4	2048	5
PASQU1860C	Mid-latitudes	Pasquale	10.58238	46.45247	2699	iButton	DS1923	26/07/2018 04:00	02/07/2019 08:00	4	2048	5
PASQU1965D	Mid-latitudes	Pasquale	10.58377	46.45311	2702	iButton	DS1923	26/07/2018 04:00	02/07/2019 08:00	4	2048	5
PASQU1986A	Mid-latitudes	Pasquale	10.58529	46.45171	2683	iButton	DS1923	26/07/2018 04:01	02/07/2019 08:01	4	2048	5
PASQU2010A	Mid-latitudes	Pasquale	10.58838	46.45257	2708	iButton	DS1923	26/07/2018 04:00	02/07/2019 08:00	4	2048	5
PEI_RG1	Mid-latitudes	Peirabroc	7.413	44.12419	2465	TinyTag Plus	TGP-4500	01/08/2019 00:00	15/09/2020 23:00	0.5	18301	15
ROTMO1858E	Mid-latitudes	Rotmoosferner	11.03063	46.84001	2287	iButton	DS1923	28/07/2018 04:00	04/07/2019 08:00	4	2048	5
ROTMO1910D	Mid-latitudes	Rotmoosferner	11.03542	46.83654	2304	iButton	DS1923	28/07/2018 04:00	04/07/2019 08:00	4	2048	5
ROTMO1921E	Mid-latitudes	Rotmoosferner	11.03565	46.83582	2306	iButton	DS1923	28/07/2018 04:01	04/07/2019 08:01	4	2048	5
ROTMO1956E	Mid-latitudes	Rotmoosferner	11.03959	46.83064	2352	iButton	DS1923	28/07/2018 04:00	04/07/2019 08:00	4	2048	5
ROTMO1993A	Mid-latitudes	Rotmoosferner	11.04374	46.8256	2436	iButton	DS1923	28/07/2018 04:00	04/07/2019 08:00	4	2048	5
RUTOR1954A	Mid-latitudes	Rutor	7.00156	45.66953	2622	iButton	DS1923	06/07/2018 04:00	12/06/2019 08:00	4	2048	5
RUTOR1988A	Mid-latitudes	Rutor	7.0003	45.66855	2614	iButton	DS1923	06/07/2018 04:00	12/06/2019 08:00	4	2048	5
RUTOR2006A	Mid-latitudes	Rutor	6.99653	45.66774	2553	iButton	DS1923	06/07/2018 04:00	12/06/2019 08:00	4	2048	5
SFORZ1920	Mid-latitudes	Sforzellina	10.50915	46.35316	2773	iButton	DS1923	31/08/2018 04:01	20/06/2019 08:01	4	1760	5
SFORZ1954	Mid-latitudes	Sforzellina	10.51041	46.35198	2790	iButton	DS1923	31/08/2018 04:01	20/06/2019 08:01	4	1760	5
SFORZ1989	Mid-latitudes	Sforzellina	10.51092	46.35155	2799	iButton	DS1923	31/08/2018 04:00	20/06/2019 08:00	4	1760	5
SFORZ2003	Mid-latitudes	Sforzellina	10.51165	46.35101	2806	iButton	DS1923	31/08/2018 04:00	20/06/2019 08:00	4	1760	5
SFORZ2015	Mid-latitudes	Sforzellina	10.51191	46.3505	2817	iButton	DS1923	31/08/2018 04:00	20/06/2019 08:00	4	1760	5
SORAP1920A	Mid-latitudes	Sorapiss Centrale	12.2249	46.51351	2192	iButton	DS1923	28/07/2018 04:00	04/07/2019 08:00	4	2048	5
SORAP1980B	Mid-latitudes	Sorapiss Centrale	12.22535	46.51309	2209	iButton	DS1923	28/07/2018 04:01	04/07/2019 08:01	4	2048	5
SORAP2009A	Mid-latitudes	Sorapiss Centrale	12.22255	46.51151	2250	iButton	DS1923	28/07/2018 04:00	04/07/2019 08:00	4	2048	5
SORCEN_3	Mid-latitudes	Sorapiss Centrale	12.22125	46.5112	2271	TinyTag Plus	TGP-4017	06/07/2017 11:01	24/08/2018 08:01	1	9934	15
SOROCC_1	Mid-latitudes	Sorapiss Occidentale	12.20618	46.5141	2417	TinyTag Plus	TGP-4500	21/06/2017 16:02	23/08/2018 13:02	1	10270	15
SOROCC_2	Mid-latitudes	Sorapiss Occidentale	12.20657	46.51534	2360	TinyTag Plus	TGP-4500	21/06/2017 18:01	23/08/2018 13:01	1	10268	15
SOROCC_3	Mid-latitudes	Sorapiss Occidentale	12.21037	46.5169	2227	TinyTag Plus	TGP-4500	22/06/2017 10:01	22/08/2018 23:01	1	10238	15
SOROCC_4	Mid-latitudes	Sorapiss Occidentale	12.21102	46.51802	2176	TinyTag Plus	TGP-4500	19/09/2017 16:00	23/08/2018 13:00	0.5	16219	15

SOROCC_AB3	Mid-latitudes	Sorapiss Occidentale	12.20839	46.51629	2298	TinyTag Plus	TGP-4500	22/07/2018 10:12	23/08/2018 13:00	0.05	15417	15
TRO_F1.1	Mid-latitudes	Trobio	10.0857	46.0558	2516	TinyTag Plus	TGP-4500	15/08/2013 00:01	15/08/2014 01:01	1	8762	15
TRO_F2.2	Mid-latitudes	Trobio	10.0772	46.0616	2368	TinyTag Plus	TGP-4500	15/08/2013 00:59	14/08/2014 23:59	1	8760	15
TROBI1911A	Mid-latitudes	Trobio	10.08131	46.06047	2427	iButton	DS1923	24/07/2018 04:01	29/06/2019 08:01	4	2042	5
TROBI2006A	Mid-latitudes	Trobio	10.08576	46.0558	2516	iButton	DS1923	24/07/2018 04:07	30/06/2019 18:08	4.016667	2042	5
BR1_01	Equatorial	Broggi	-77.58202	-9.00148	4972	HOBO	8K Pendant	15/06/2019 21:00	11/06/2021 17:00	4	4362	10
BR1_02	Equatorial	Broggi	-77.58301	-9.00091	4910	HOBO	8K Pendant	15/06/2019 21:00	11/06/2021 13:00	4	4361	10
BR1_03	Equatorial	Broggi	-77.58297	-9.00092	4910	HOBO	8K Pendant	15/06/2019 21:00	11/06/2021 13:00	4	4361	10
BR2_01	Equatorial	Broggi	-77.58529	-9.00237	4727	HOBO	8K Pendant	15/06/2019 21:00	11/06/2021 13:00	4	4361	10
BR2_02	Equatorial	Broggi	-77.58519	-9.00241	4727	HOBO	8K Pendant	15/06/2019 21:00	11/06/2021 13:00	4	4361	10
BR2_03	Equatorial	Broggi	-77.5856	-9.00296	4698	HOBO	8K Pendant	15/06/2019 21:00	11/06/2021 13:00	4	4361	10
BR3_01	Equatorial	Broggi	-77.58821	-9.0041	4619	HOBO	8K Pendant	15/06/2019 21:00	11/06/2021 13:00	4	4363	10
BR3_02	Equatorial	Broggi	-77.58824	-9.00409	4619	HOBO	8K Pendant	15/06/2019 21:00	11/06/2021 12:00	4	4357	10
BR3_03	Equatorial	Broggi	-77.58879	-9.00423	4621	HOBO	8K Pendant	15/06/2019 21:00	11/06/2021 08:00	4	4354	10
BR4_01	Equatorial	Broggi	-77.58935	-9.00513	4625	HOBO	8K Pendant	15/06/2019 21:00	11/06/2021 05:00	4	4359	10
BR4_02	Equatorial	Broggi	-77.58933	-9.00517	4618	HOBO	8K Pendant	15/06/2019 21:00	11/06/2021 05:00	4	4359	10
BR4_03	Equatorial	Broggi	-77.59055	-9.0054	4594	HOBO	8K Pendant	15/06/2019 21:00	11/06/2021 05:00	4	4359	10
BRLIA_01	Equatorial	Broggi	-77.59979	-9.0134	4465	HOBO	8K Pendant	15/06/2019 21:00	13/06/2021 09:00	4	4372	10
BRLIA_02	Equatorial	Broggi	-77.60273	-9.01144	4436	HOBO	8K Pendant	15/06/2019 21:00	13/06/2021 09:00	4	4372	10
U1_01	Equatorial	Uruashraju	-77.32208	-9.59482	4669	HOBO	8K Pendant	23/06/2019 00:00	26/05/2021 20:00	4	4158	10
U1_02	Equatorial	Uruashraju	-77.32305	-9.59508	4618	HOBO	8K Pendant	23/06/2019 00:00	26/05/2021 20:00	4	4158	10
U1_03	Equatorial	Uruashraju	-77.32308	-9.59511	4618	HOBO	8K Pendant	23/06/2019 00:00	26/05/2021 20:00	4	4158	10
U2_01	Equatorial	Uruashraju	-77.32431	-9.59607	4593	HOBO	8K Pendant	23/06/2019 00:00	26/05/2021 20:00	4	4158	10
U2_02	Equatorial	Uruashraju	-77.3243	-9.59606	4608	HOBO	8K Pendant	23/06/2019 00:00	26/05/2021 20:00	4	4158	10
U2_03	Equatorial	Uruashraju	-77.32484	-9.59715	4601	HOBO	8K Pendant	23/06/2019 00:00	26/05/2021 20:00	4	4158	10
U3_01	Equatorial	Uruashraju	-77.32574	-9.59851	4586	HOBO	8K Pendant	23/06/2019 00:00	26/05/2021 20:00	4	4158	10
U3_02	Equatorial	Uruashraju	-77.32575	-9.59849	4586	HOBO	8K Pendant	23/06/2019 00:00	26/05/2021 20:00	4	4158	10
U3_03	Equatorial	Uruashraju	-77.32655	-9.59882	4571	HOBO	8K Pendant	23/06/2019 00:00	26/05/2021 20:00	4	4158	10
U4_01	Equatorial	Uruashraju	-77.32731	-9.59945	4536	HOBO	8K Pendant	23/06/2019 00:00	26/05/2021 20:00	4	4158	10
U4_02	Equatorial	Uruashraju	-77.32731	-9.59947	4536	HOBO	8K Pendant	23/06/2019 00:00	26/05/2021 20:00	4	4158	10
U4_03	Equatorial	Uruashraju	-77.32671	-9.59921	4563	HOBO	8K Pendant	24/06/2019 00:00	26/05/2021 20:00	4	4152	10
ULIA_01	Equatorial	Uruashraju	-77.33284	-9.6083	4361	HOBO	8K Pendant	10/01/2021 00:00	26/05/2021 20:00	4	822	10
ULIA_02	Equatorial	Uruashraju	-77.33288	-9.60827	4361	HOBO	8K Pendant	10/01/2021 00:00	26/05/2021 20:00	4	822	10

ULIA_03	Equatorial	Uruashraju	-77.33326	-9.60895	4352	HOBO	8K Pendant	10/01/2021 00:00	26/05/2021 20:00	4	822	10
Y1_01	Equatorial	Yanamarey	-77.27258	-9.65607	4680	HOBO	8K Pendant	15/06/2019 21:00	02/06/2021 20:00	4	4291	10
Y1_02	Equatorial	Yanamarey	-77.2726	-9.65611	4680	HOBO	8K Pendant	15/06/2019 21:00	02/06/2021 20:00	4	4291	10
Y1_03	Equatorial	Yanamarey	-77.27188	-9.65687	4694	HOBO	8K Pendant	15/06/2019 21:00	02/06/2021 20:00	4	4291	10
Y2_01	Equatorial	Yanamarey	-77.27354	-9.65896	4641	HOBO	8K Pendant	02/06/2019 21:00	02/06/2021 20:00	4	4369	10
Y2_02	Equatorial	Yanamarey	-77.27353	-9.65898	4641	HOBO	8K Pendant	15/06/2019 21:00	02/06/2021 20:00	4	4291	10
Y2_03	Equatorial	Yanamarey	-77.27278	-9.65843	4659	HOBO	8K Pendant	15/06/2019 21:00	02/06/2021 20:00	4	4291	10
Y3_01	Equatorial	Yanamarey	-77.27613	-9.66112	4627	HOBO	8K Pendant	15/06/2019 21:00	02/06/2021 20:00	4	4291	10
Y3_02	Equatorial	Yanamarey	-77.27611	-9.66112	4627	HOBO	8K Pendant	15/06/2019 21:00	02/06/2021 20:00	4	4291	10
Y3_03	Equatorial	Yanamarey	-77.27729	-9.66099	4608	HOBO	8K Pendant	15/06/2019 21:00	02/06/2021 21:00	4	4309	10
Y4_01	Equatorial	Yanamarey	-77.27809	-9.66132	4605	HOBO	8K Pendant	15/06/2019 21:00	02/06/2021 21:00	4	4309	10
Y4_02	Equatorial	Yanamarey	-77.27809	-9.66127	4605	HOBO	8K Pendant	15/06/2019 21:00	11/01/2021 21:00	4	3457	10
Y4_03	Equatorial	Yanamarey	-77.27712	-9.66167	4604	HOBO	8K Pendant	15/06/2019 21:00	02/06/2021 21:00	4	4309	10
YLIA_01	Equatorial	Yanamarey	-77.28569	-9.66883	4420	HOBO	8K Pendant	15/06/2019 21:00	13/06/2021 21:00	4	4375	10
YLIA_02	Equatorial	Yanamarey	-77.28546	-9.66868	4423	HOBO	8K Pendant	15/06/2019 21:00	13/06/2021 21:00	4	4375	10
