# Supplementary material for: Heterogeneous changes of soil microclimate in high mountains and glacier forelands
Source: Nat Commun. 2023 Aug 31;14:5306. doi: 10.1038/s41467-023-41063-6 (PMC10471727; doi:10.1038/s41467-023-41063-6)
Supplement: Supplementary file 3 — Description of Additional Supplementary Files [file 41467_2023_41063_MOESM3_ESM.pdf]

Description of Additional Supplementary Files

File Name: Supplementary Software 1
Description: R code used to generate temperature projections at the global scale
